# Fine-Scale Population Structure of Blue Whale Wintering Aggregations in the Gulf of California

**DOI:** 10.1371/journal.pone.0058315

**Published:** 2013-03-07

**Authors:** Paula Costa-Urrutia, Simona Sanvito, Nelva Victoria-Cota, Luis Enríquez-Paredes, Diane Gendron

**Affiliations:** 1 Laboratorio de Ecología Molecular, Facultad de Ciencias Marinas, Universidad Autónoma de Baja California, Baja California, Mexico; 2 Elephant Seal Research Group, Sea Lion Island, Falkland Islands; 3 Laboratorio de Ecología y Epidemiología Molecular, Escuela de Ciencias de la Salud, Universidad Autónoma de Baja California, Baja California, Mexico; 4 Laboratorio de Ecología de Cetáceos y Quelonios, Centro Interdisciplinario de Ciencias Marinas, Instituto Politécnico Nacional, Mexico City, Mexico; Auburn University, United States of America

## Abstract

Population differentiation in environments without well-defined geographical barriers represents a challenge for wildlife management. Based on a comprehensive database of individual sighting records (1988–2009) of blue whales from the winter/calving Gulf of California, we assessed the fine-scale genetic and spatial structure of the population using individual-based approaches. Skin samples of 187 individuals were analyzed for nine microsatellite loci. A single population with no divergence among years and months and no isolation by distance (Rxy = 0.1–0.001, p>0.05) were found. We ran two Bayesian clustering methods using Structure and Geneland softwares in two different ways: 1) a general analysis including all individuals in which a single cluster was identified with both softwares; 2) a specific analysis of females only in which two main clusters (Loreto Bay and northern areas, and San Jose-La Paz Bay area) were revealed by Geneland program. This study provides information indicating that blue whales wintering in the Gulf of California are part of a single population unit and showed a fine-scale structure among females, possibly associated with their high site fidelity, particularly when attending calves. It is likely that the loss of genetic variation is minimized by male mediated gene flow, which may reduce the genetic drift effect. Opportunities for kin selection may also influence calf survival and, in consequence, have a positive impact on population demography in this small and endangered population.

## Introduction

Detecting population structure in taxa characterized by continuous distribution in environments without well-defined geographical barriers is one of the most challenging problems in population genetics. This applies to the conceptual definition of populations, but also represents a challenge for conservation and wildlife management. Following the evolutionary paradigm proposed by [1] we consider a population as “a group of individuals of the same species living in close enough proximity that any member of the group can potentially mate with any other member”. However, fine-scale genetic structure may be a confounding factor if the population shows distinct levels of genetic stratification as heterogeneity are among adjacent breeding groups or spatial segregated kin selection [2].

Many aspects of life history, as well as several environmental factors, can influence the structure of populations and, therefore, the individual’s dispersal potential alone does not always allow predictions about the gene flow among populations. This is especially true for cetaceans, for which movements are not limited by physical barriers. Although most cetacean species show wide distributions and high potential for dispersal, their populations usually exhibit some kind of structure [3], [4], [5], [6], [7], that may depend on the local distribution of resources and/or their social system [8].

Female philopatry and migration patterns are known to have a strong influence on population structure in large mammals [8], [9], as observed in humpback whales, *Megaptera novaeangliae* [10], northern and southern right whales *Eubalaena glacialis* and *E. australis* respectively [11], [12], [13], grey whales, *Eschrichtius robustus* [14], [15], and sperm whales, *Physeter macrocephalus*, [16], [17]. Recently, maternal site fidelity was also suggested for the Antarctic blue whales (*Balaenoptera musculus intermedia*), that showed divergence in mitochondrial DNA and microsatellite markers among the six feeding grounds in the Antarctic [18]. In general, baleen whale females disperse less than males [8], but few studies addressed the fine-scale population structure, probably because of the time and effort required to obtain long-term serial records of individually recognized whales.

The blue whale (*Balaenoptera musculus*) is a cosmopolitan species, with both coastal and oceanic distribution. Like most baleen whales, this species migrates between feeding grounds, located in areas at high latitudes, and calving grounds at mid and low latitudes [19]. This species was one of the main targets of commercial whaling and, due to its reduced population size and slow recovery rate, it is still considered endangered [20]. The largest known remnant population, between 2000 and 3000 blue whales [21], inhabits the eastern North Pacific. This population appears to be separated from populations in the central and western North Pacific, as suggested from differences in call types [22], [23]. The link between the blue whales sighted off California, their main feeding area in summer, and those in the Gulf of California (GC) in winter has long been demonstrated by photo-recapture evidences [24] and also well-illustrated through the movements of individuals tracked with satellite tags [25]. Around 300 blue whales are estimated to winter annually in the GC, and then migrate northwest along the Pacific coast of Baja California, following the seasonal shift of marine productivity around the peninsula [26]. Long-term surveys and photo-identification records in the southwestern GC showed seasonal residence periods that ranged widely from few days to over 70 days [26]. This study has also pointed out that the GC is an important calving and feeding ground for this population, thus making it a promising site where to study the fine-scale population structure of this species.

A molecular analysis of Southern Hemisphere blue whale feeding aggregations showed that three geographic areas (Southeast Pacific, Indian Ocean and Antarctica) were occupied by distinct populations, and in each of them no structure was found probably due to small sample sizes [27]. More recently, a low but significant divergent structure was found among the six feeding grounds in the Antarctic Ocean [18]. However, a larger sample size was used to analyze the Perth Canyon and Bonney Upwelling, two Australian feeding grounds, but no population structure was found [28].

In this paper we investigated the population structure of blue whales in the GC, the only studied calving ground for this species using an extensive data base of individual sighting histories obtained during the last 22 years [29]. Being interested only in the fine-scale structure of a small, localized, but continuously distributed aggregation, we selected microsatellites as the optimal marker to carry out our study [30], [31]. We expected to find some structuring, at least among females, due to the high female site fidelity and philopatry found in calving grounds of other baleen whales [8], [9], [15], [18], and to the observed bias toward females in the sex ratio of blue whales sampled in the GC (females/males = 1.5/1) [26].

## Methods

### Study Area

**Figure 1:**
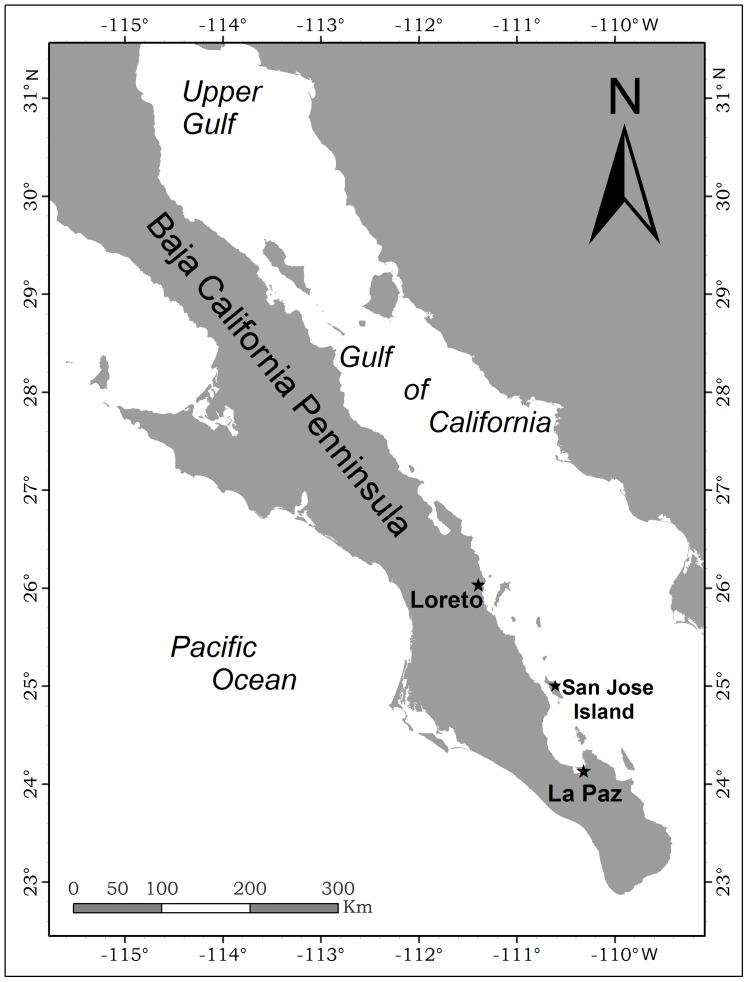
Map of the study area.

The GC is a narrow internal sea that separates Baja California Peninsula from the Mexican mainland, located between 23° to 31°N and between 107° to 115°W in the Northeast Pacific Ocean (Figure 1). It is 1,120 km long and 150 km wide on average, with an area of about 200,000 square kilometers [32], considered highly productive from December to June [33].

### Sample Collection

We conducted 2–6 day-long surveys between January and May 1988–2009, covering the area between the cities of La Paz and Loreto (Figure 1). Moreover, longer surveys were conducted covering the whole GC in 2005 and the Upper GC in 2006. Once a whale was sighted, the GPS positions were recorded and photographs for individual identification were taken [34]. We used the CICIMAR photo-ID catalogue categorized by the dorsal fin shapes and the body pigmentation patterns to identify the animals [29].

From 1997 to 2009 skin-blubber biopsies were collected with a similar system as described in Lambersten [35], using a dart of 7 mm of diameter and 40 mm long stainless steel core sampler with three internal, inward-facing barbs. The dart is screwed in the tip of an arrow designed with a stopper that limits the depth of penetration and makes the arrow rebound out with the sample in it. The arrow was fired from a 68 kg crossbow at a distance of about 10 m from the whale and retrieved once it floated. Skin-blubber biopsies were extracted from the dart using sterilized stainless tweezers. In order to prevent contamination between samples and infection to the animals, the core sampler was sterilized before each biopsy attempt by immersion in a 50% chlorine solution, then transferred to a 70% ethanol solution, exposed for 10 s to a blowtorch flame and finally wrapped in aluminium foil.

We avoided duplicating biopsy samples within and between seasons by comparing newly photographed whale (digital camera viewing) with those included in a field catalogue of biopsied whales. The biopsies collected from 1997 to 2001 were preserved in 20% dimethylsulfoxide (DMSO) saturated with table salt [36], thereafter samples were immediately frozen in liquid nitrogen. It is important to clarify that biopsy-sampling procedure started in 1997, but several of the biopsied individuals in our data base have long sighting histories; thus all the sightings from each biopsied individual were used in this study.

### Ethics Statement

Photo-identification and biopsy samples were collected under the annual research permits () issued by the Secretaria de Medio Ambiente Recursos Naturales y Pesca (180796-213-03, 071197-213-03, DOO 750-00444/99, DOO.0-0095, DOO 02.-8318) from 1997 to 2001, and by the Dirección General de Vida Silvestre, Secretaría de Medio Ambiente y Recursos Naturales (SGPA/DGVS-7000, 00624, 01641, 00560, 12057, 08021, 00506, 09760) from 2002 to 2009, which now represents the only approved Government institution to issue research permits on endangered species in Mexico. By issuing these annual research permits they approved the number of blue whale skin-blubber biopsies collection for each year of the permit. Our institution does not have an Animal Ethics Committee; therefore no information is given on that aspect.

### Sample Processing and Analysis

Total DNA was extracted from skin using the standardized protocol [37]. We carried out genotyping at 19 microsatellite loci on a subset of 92 samples to evaluate polymorphisms. We found low variability (two to four alleles) for nine microsatellites: AC45, CAAA74, AC82, GT122, GT129, GT227, CA141, GATA53 [38], DlrFC17 [39]. The 10 most polymorphic and informative loci were chosen for further analyses (Table 1) [40], [41], [42]. We performed PCR reactions with 30 ng of DNA template and the following reagent concentrations: 22 mM Tris-HCl pH 8.4, 55 mM KCl, 1.2 U DNA *Taq* Polymerase (Invitrogen™), 0.5 µM of each primer (forward primers were labeled with a fluorescent dye, 6-FAM®, VIC® or NED®, of Applied Biosystems), 0.2 mM dNTPs and MgCl_2_ concentration as specified in Table 1. The following PCR cycling profile was used: initial denaturation step at 94°C for 2 min, followed by 7 cycles consisting of denaturation at 96°C for 20 sec, annealing at AT°C_1_ (see Table 1) for 30 sec and extension at 72°C for 30 sec, followed by 25 cycles of denaturation at 95°C for 30 sec, annealing at AT°C_2_ (see Table 1) for 30 sec and extension at 72°C for 30 sec, and a final extension step at 72°C for 15 min. We resolved PCR products on an ABI Prism 310 genetic analyzer using the GeneScan™ 600 LIZ® size standard (Applied Biosystems) and included non-template PCR controls to check for cross-contamination and replicates (nine percent of the samples) to evaluate the reproducibility and genotyping errors. We processed the raw ABI files with GeneMarker® 1.9 software (SoftGenetics) to obtain the genotypes, validated the allele binning with FlexiBin software [43], and checked the genotypes for null alleles and genotyping errors with Micro-checker v.4.0.7 [44]. The gender of each individual was identified by PCR amplification of ZFX and ZFY genes [45].

**Table 1:** Statistics of the nine microsatellite loci.

Locus	MgCl_2_ (mM)	AT_1_/AT_2_(°C)	Na	Size	H_O_	H_E_
GATA98^a^	4.7	51°/52°	9	74–120	**0.69**	**0.73**
GT541^b^	3.1	56°/55°	9	79–99	0.7	0.76
AC137^b^	3.1	55°/54°	9	91–119	**0.64**	**0.66**
GT023^c^	3.1	60°/58°	6	114–124	0.77	0.76
CA232^b^	3.1	56°/55°	8	142–168	0.65	0.65
AC087^b^	3.1	56°/55°	12	165–180	0.83	0.83
EV037^d^	3.9	51°/52°	8	172–194	0.58	0.58
GATA417^a^	3.1	56°/55°	13	174–226	0.83	0.83
CA234^b^	3.1	55°/54°	13	191–215	0.91	0.88
Mean			9.6		0.74	0.74
SD			2.4		0.03	0.09

At = Annealing temperature (subscript _1_ and _2_ correspond to the first and second PCR cycle). Na = Number of alleles per locus, Size = observed range in fragment size in base pairs (bp), Observed (H_O_) and Expected (H_E_) heterozygosity per locus. H_O_ deficiency loci are highlight in bold (p<0.05).

a 36 Palsbøll et al., 1999,

b 34 Bérubé et al. 2005,

c 37 Bérubé et al. 200,

d 38 Valsecchi and Amos 1996.

### Data Analysis

#### Heterozygosity, hardy-weinberg equilibrium and linkage disequilibrium

We calculated the number of alleles per locus, the observed (H_O_) and the expected (H_E_) heterozygosity and with Arlequin 3.1 [46]. We tested linkage disequilibrium (LD) between loci, deviations from Hardy-Weinberg equilibrium (HWE) for each locus, and global deviation from HWE for all loci, using the MCMC method (10000 iterations) [47] implemented in Genepop 4.0 [48]. We corrected the significance levels of HWE and LD test for multiple comparisons with sequential Bonferroni adjustments [49]. We calculated F_IS_ coefficient in Fstat 2.9.3.2 [50].

#### Temporal variation in population structure

Based on photo-recapture records, we defined groups (more than 10 individuals) according to field season when individuals were sighted (hereafter “years”), and to the month and year of sightings (hereafter “month-year*”*). As a result of this criterion, the 1997–2009 period for the “years” category, and the 1999–2009 period for the “month-year” category were analyzed (Table 2). Additionally, individuals were assigned to one of three categories based on the number of years (consecutive or not) in which we observed them (“sighting frequency groups”): i) occasional (observed only in one year), ii) frequent (two-four years), and iii) highly frequent (five years or more).

**Table 2:** Blue whale grouping criteria for population structure testing at the temporal scale.

Grouping Criteria	Groups	Size	Sex ratio	HWE	R_ST,_ F_ST_
Years	13	16–61		**2009 group: p = 0.0014**	R_ST_ = 0–0.01, p>0.05^a^
		mode = 39, SD = 12	X^2^ = 4.5, p = 0.9, df = 12	Remaining Groups:p = 0.3–0.008	F_ST_ = 0–0.03, p>0.05^a^
				adjusted B p = 0.004,13 test	
Month-year	23	10–36	**February:X^2^ = 43.1, p = 0.03, df = 9**	**March 2009:p = 0.0018**	R_ST_ = 0.0001–0.03, p = 0.01–0.9
		mode = 11, SD = 8	March: X^2^ = 15, p = 0.07, df = 10	Remaining Groups:p = 0.3–0.008	F_ST_ = 0.0001–0.001, p = 0.03–0.9
			April: X^2^ = 0.6, p = 0.6, df = 1	adjusted B p = 0.0022,23 test	adjusted B-Yp = 0.008
Sighting frequency	3	39–69	X^2^ = 0.68, p = 0.8, df = 2	p = 0.02–0.2,	R_ST_ = 0–0.001, p>0.05^a^
		mode = 39, SD = 17		adjusted B p = 0.01	F_ST_ = 0–0.0004, p>0.05^a^

Grouping criteria: see Methods. Groups = number of groups of the temporal structure analyses. Size = range, mode and standard deviations of groups. Sex ratio = sex ratio of the group compared to overall sex ratio (1.41∶1) in the population. HWE = Hardy-Weinberg equilibrium. R_ST_ and F_ST_ = range values of pairwise R_ST_ and F_ST_ comparisons among groups. Adjusted B and B-Y p-values refer to the adjusted p-values after sequential Bonferroni and Benjaminy-Yekutiely correction respectively.

a denotes all p-values were >0.05. Significant results are in bold.

All above grouping criteria (years, month-years and sighting frequency) were tested for departure from our population sex ratio (see results) with chi-square test. We tested deviation from HWE and calculated the fixation indices- R_ST_ [51] and F_ST_ [52] of genetic differentiation between groups with Arlequin 3.1. Adjustments for multiple comparisons were made for dependent (Benjamini-Yekutieli, [53]) and independent samples (sequential Bonferroni’s, [49]) for HWE, F_ST_ and R_ST_. We used the correction for dependent samples [53] for year and month-year groups, since the same individual could be included in more than one group, and sequential Bonferroni’s [49] adjustment for sighting frequency groups as they are independent groups.

#### Isolation by distance (IBD)

To verify the presence of isolation by distance we carried out an individual-based Mantel test, both on the complete dataset (females and males pooled) and on females only, using the kinship coefficients R_QG_ [54] and R_RL_ [55] as measures of genetic distance. A geographic distance matrix was made based on geometric means of the GPS fixes, for individuals recorded in more than one position (68%), which can be considered a rough estimate of the center of individual activity [56]. The P-value for the correlation coefficient was calculated with a permutation test (10000 replicates) as implemented in Genalex 6 [57].

#### Bayesian clustering

We carried out two Bayesian clustering analysis, both on the complete dataset (females and males pooled) and on females only. In the first, we used the admixture model with correlated allele frequency implemented in Structure 2.3 [58]. The number of clusters (K) ranged from 1 to 8, and for each K we run 10 independent MCMC runs with 10^6^ iterations, following a burn-in period of 60000 iterations. The mean values of the 10 runs for each K were reported, and we chose the K value with the highest probability (Ln P(D)) as the best estimated number of clusters. In the second analysis, we used the mixture model with correlated allele frequency and free Voronoi tessellation [59] implemented in the R package Geneland [60]. This program uses geo-referenced multi-locus genotyped individuals to probabilistically assign them to a cluster. We carried out 25 independent runs using 10^6^ iterations and K_max_ = 10. Each set of 25 runs were ranked using the posterior probability of each, and we post-processed the best 10 runs. We obtained the K value from the mode value of these 10 runs, and reported the best posterior probabilities of this mode. For post-processing a burn-in period of 60000 iterations and a 800×200 pixel resolution for x and y axis were used respectively. The geo-referencing of each individual used in this analysis was the same as in the individual-IBD analysis (see above). The range of some blue whale movements can extend as much as the whole study area [26] and this complicates the estimation of the position uncertainty. To calculate the uncertainty in the positioning of individuals that was inputted in Geneland, we did a four-step analysis: 1) using the first daily positions of the individuals resighted between 1988- 2009; 2) estimating the 95% area of activity with the Kernel Density Estimator (KDE) [61] implemented in the Home Range Tool extension [62] of ArcGIS 9.3 (ESRI); 3) calculating the kernel bandwidth with the Least Square Cross Validation method; 4) using the square root of the mean individual KDE area as measure of uncertainty. We used the maximum number of locations per individuals that showed no positive correlation with the estimated size area. As a result, twenty individuals (n = 17 females, n = 3 males) were used to estimate the KDE individual utilization area. The number of locations of these individuals ranged between 11 and 44 per individual, and no linear correlation was found with the estimated KDE (Pearson = 0.05, p = 0.9). The mean KDE_95%_ was 3197.6 km^2^, thus the uncertainty value used was 57 Km.

In order to verify if clusters obtained were artifacts of the models we analyzed HWE and divergence between them, using clusters with at least five individuals [59]. We calculated R_ST,_ F_ST_ and F_IS_ between and within clusters respectively. For both models we performed 10 preliminary runs in order to know the maximum K value and the length of the chain that should be used so as not to affect the results.

## Results

### Genetic Variation

PCR conditions and statistics on the allelic variation of each locus are presented in Table 1. The locus GATA28 showed evidence for the presence of null alleles and we decided to exclude this locus from the analyses. Two loci departed from HWE, GATA98 and AC137 (Table 1), though there was no global deviation from HWE when all loci were considered (x^2^ = 28.8, p = 0.053). No pair of loci was in linkage disequilibrium (observed p = 0.0024–0.9, sequential Boferroni p = 0.0014, 36 test). The inbreeding coefficient was close to zero (F_IS_ = 0.007, p = 0.3), and the mean observed (H_O_) and expected (H_E_) heterozygosity were equal (H_O_ = 0.74, H_E_ = 0.74) (Table 1).

A total of 187 blue whale samples were analyzed for 9 microsatellite loci. We obtained a 100% match in genotyping of 5 duplicated samples that were classified as different individuals by photo-ID. These 10 samples were excluded and we carried out all further analyses on 177 whales (Females = 99, Males = 70, Not sexed = 8). In accordance to a previous study [26], our blue whale sex ratio (females/males = 1.41/1) was skewed towards females.

### Temporal Variation of the Population Structure

#### Years

The number of individuals observed ranged from 16, in 1998, to 61 in 2009 (Table 2). We found no deviation from the 1.41/1 (female/male) population sex ratio among years (Table 2). We found a significant deviation from HWE only in 2009 (observed p = 0.0007, Benjamini-Yekutieli p = 0.004, 13 test). No significant divergence was found among years (Range: R_ST_ = 0–0.01, p>0.05, F_ST_ = 0–0.03, p>0.05).

#### Months-year

This analysis involved 23 groups observed in February (n = 10 groups), March (n = 11) and April (n = 2). We found a significant deviation from the 1.41/1 population sex ratio towards females in February (female/male = 3/1; Table 2). A significant departure from HWE was found only in March-2009 group. Out of a total of 253 month-group pairwise comparisons, we found small but significant R_ST_ and F_ST_ in nine comparisons_,_ but not after we applied the Benjamini-Yekutieli correction (Table 2).

#### Sighting frequency

Sixty-nine individuals were sighted as occasional, 69 as frequent and 39 as highly frequent. We found no deviation from the 1.41/1 population sex ratio among groups, neither deviation from HWE after sequential Bonferroni correction, nor significant divergence among groups (Table 2). Since no evidence of genetic structure in a temporal basis was found, all samples were grouped for the spatial genetic analysis.

### Spatial Structure

#### Isolation by distance

No evidence of IBD was found in individual-based Mantel tests performed on the complete data set (Rxy_QG_ = 0.01 p = 0.3, Rxy_RL_ = 0.01 p = 0.17), nor in females only data set (Rxy_QG_ = 0.05 p = 0.24, Rxy_RL_ = 0.05 p = 0.5).

#### Bayesian clustering analysis

**Figure 2:**
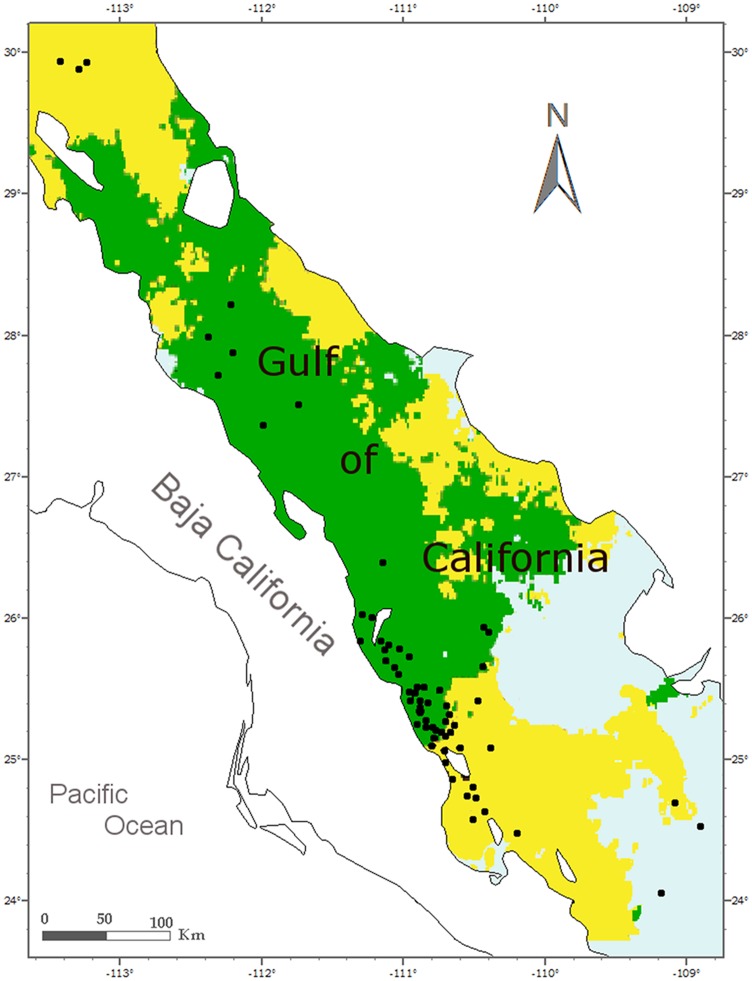
Results of the Structure model **fitting.** K = number of population. Ln P(D) = logarithm of the data probability obtained for complete (a) and female (b) data set. Highest posterior probability in both cases is for k = 1.

**Figure 3:**
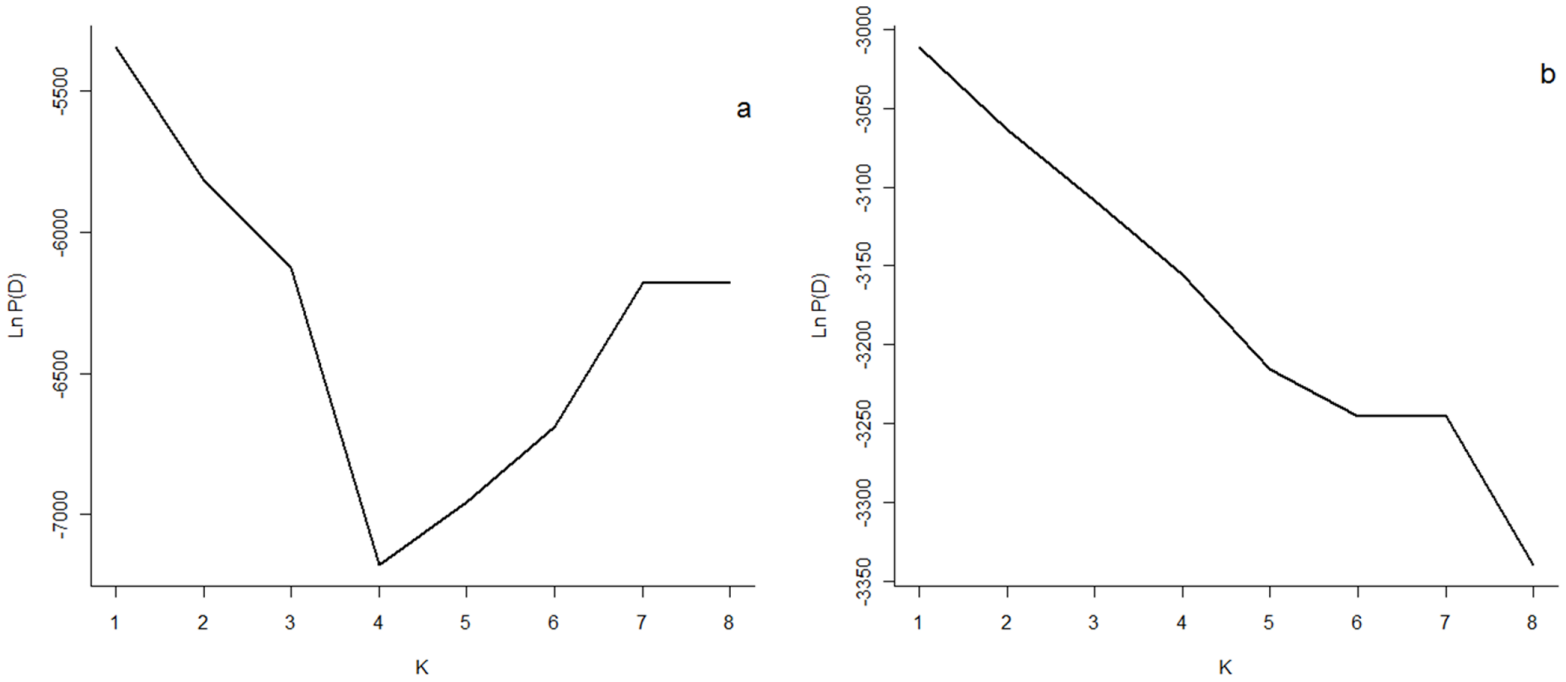
Map of the mode posterior probabilities obtained with the Geneland model. Estimated clusters of blue whales in the Gulf of California are shown in different colours. Green cluster represent Loreto cluster and yellow cluster represent San Jose- La Paz cluster. Dots represent the geographic centroid of individual female blue whale sightings.

Using the admixture model with correlated allele frequencies implemented in Structure software, we found only one cluster (K = 1) in both analyses (complete and females only data set, Figure 2). All individuals were admixed and assignment values were close to 0.5. This indicates the program is assigning individuals randomly to K populations, owing to the lack of underlying population structure [63]. Using the mixture model with correlated allele frequency and free Voronoi tessellation, implemented in Geneland, only one cluster for the complete dataset was found. In contrast, when the same model was run only for females, most individuals (96%) were assigned to two clusters with low but significant divergence (Table 3). Although one of the clusters is mainly represented in the central portion (green cluster, herein Loreto cluster) and the other in the south (yellow cluster, herein San Jose- La Paz cluster), no clear homogenous distribution of these clusters was found. Individuals from the yellow cluster (named San José-La Paz cluster) were also found in the northern GC, which suggests that individuals from both clusters do not show a complete segregation among these areas (Figure 3).

**Table 3:** Results of the fitting of the Bayesian clustering model with Geneland program.

Data set	K	Individuals per K	Divergence (R_ST,_ F_ST_)	Inbreeding (F_IS_)
Complete	3(1)	1 = 171,2 = 3,3 = 3		
Females	3(2)	1 = 66, 2 = 29, 3 = 4	**1–2: R_ST_ = 0.01, p = 0.01**	**1:F_IS_ = 0.01, p = 0.3**
			**1–2: F_ST_ = 0.008, p = 0.02**	**2: F_IS_ = 0.03, p = 0.07**

K = number of inferred clusters; in brackets are the number of K with significant R_ST_ and F_IS_ close to zero. Individual per K = number of individuals assigned to each K. R_ST_ and F_ST_ = pairwise divergence among K, F_IS_ = inbreeding coefficient per K. Significant divergence and inbreeding close to zero are in bold, p = p-value at 95% confidence.

## Discussion

In this study we investigated the fine-scale population structure of blue whales that winter in the GC. Our work provides information that suggests these blue whales are part of a single population unit. This is supported by all analyses; temporal grouping criteria, isolation by distance and Bayesian clustering approach, conducted on complete data sets. However, structure among females was found using the Geneland model.

The mean observed heterozygosity of blue whales in the GC (H_O_ = 0.74) was similar to values reported for this species in the South Pacific (Ho = 0.72) and the Antarctic (Ho = 0.75) Oceans [27], and higher than those found in Australian aggregations (H_O_ = 0.66 in Perth Canyon, Western Australia; Ho = 0.59 in Bonney Upwelling, Southern Australia) [28]. Despite the smaller estimated blue whale population size in the GC (n = 283, %CV = 48.4) [26], the observed diversity and the inbreeding coefficient (F_IS_ close to zero) suggest a panmictic population.

The lack of a clear population structure for the whole data set found in this study agrees with the lack of structure found in blue whales of the southern hemisphere [28]. It also agrees with the results on bioacoustic studies that suggest the presence of a single population in the Northeast Pacific, extending from Vancouver to the Dome of Costa Rica, and possibly beyond Ecuador [22], [23]. Our analysis based on individual sighting frequencies also supports one Northeast Pacific genetic stock, since occasional individuals (sighted only one year) that possibly use other wintering grounds are not genetically divergent from the frequent (two-four years) and very frequent (five years or more) individuals. Movements of blue whales from California to the Costa Rica Dome during winter have been reported [25], but whales were also found year-round in that upwelling-enriched area [64]. Thus, some gene flow between the northern and southern Pacific blue whales is plausible. In this context, if some of our occasional individuals also use the Costa Rica Dome wintering area, they may guarantee enough gene flow in the Northeast Pacific population so as to result in a single homogenous genetic stock. Additionally, certain amount of gene flow further south could not be discarded.

Although we found no genetic divergence among individuals of different years and months-year, we observed some signs of a temporal segregation of females. Overall, there is a bias in the sex ratio in the GC toward females, which becomes stronger in February in all years analyzed (month-year group, Table 2). This higher proportion of females observed in February may depend on their reproductive status. Births likely occur between January and March [26], and late-pregnant females could be the earliest individuals to reach the GC to give birth, producing the bias toward females at the beginning of the season. Segregating migration has been shown in humpback whales, although in that species late-pregnant females is the last class of individuals to arrive to the wintering ground [65]. Blue whales are using the GC also as a feeding ground, where they feed principally on the abundant euphausiid, *Nyctiphanes simplex* [66]. Lactating females are often emaciated [26] and likely have higher nutritional requirements. Therefore, pregnant females may match the birth period with the period of highest density of their main prey, which occurs in February-March [67].

Although we found no genetic sub-structuring of the population when analyzing the whole data set, we obtained two clusters when analyzing only females with Geneland. Differences in the results between the two Bayesian approaches cannot be explained straightforward, but it has been reported that admixture models fail to find clusters in shallow divergent populations [68], and especially the Structure model tends to gather individuals into the largest cluster, suggesting an unreal lack of structure [69]. By using a Bayesian model that takes into account spatial information, the analysis has proven to be more powerful [60]. Even though this approach looks promising, our result should be interpreted with caution. When applied to wild populations, Bayesian spatial clustering models may infer a wrong number of clusters [63], [68], [70], [71]. To reduce this risk we ruled out isolation by distance that may greatly affect Bayesian clustering inference [70]. We compared the genetic divergence and the inbreeding between and within the clusters respectively, to test if they were real or artifact, and found a small but significant divergence. Furthermore, we used an uncertainty value produced by the analysis of individual utilization area of blue whales in the GC.

As mentioned above, it has been reported that Structure models fail to find structure when F_ST_ values are low (F_ST_<0.02) [63], [68]. According to this, Structure model fails to find a structure among the six Antarctic blue whale feeding grounds defined upon whaling records (F_ST_ = 0.005 p = 0.031) [18]. Thus, it is not surprising not to find any structure with Structure analysis under the low divergence among clusters found in this study (R_ST_ = 0.01, p = 0.01, F_ST_ = 0.008, p = 0.02). The spatial distribution of the genetic clusters was not completely homogeneous, as expected in very mobile animals, suggesting that the genetic segregation is only partially matched by ecological segregation. Moreover, the low values of R_ST_ and F_ST_ indicate that gene flow is maintained between clusters, allelic frequencies are correlated among them, and the recognition of clusters may be difficult [59], [72], [73].

The uncertainty in spatial position is an important parameter of the Geneland model, and allows to take into consideration both the measurement error (e.g., coordinates recorded with low precision), and the process error (e.g., very large range of movements). Our uncertainty value was calculated mainly from females’ individual utilization areas and, therefore, the higher power in detecting clusters with data on females may just be an effect of the better representation of their spatial structure.

Fine-scale population structure has been reported in various mammalian species. In most cases, female natal philopatry generates aggregated grouping patterns, and this produced some level of genetic differentiation [2], [31], [74]. In a worldwide sperm whale population structure analysis [17] no microsatellite divergence was found at large spatial scale (oceans), but significant genetic heterogeneity was found between female social groups, suggesting the presence of greater relatedness within groups than between them, and a potential role of kin selection in sperm whale evolution. The blue whales social structure is poorly understood [26], but overall our results indicate 1) no temporal divergence but seasonal segregation among females, 2) no spatial divergence from the complete data set and the inbreeding coefficient close to zero and 3) no homogeneous distribution of Geneland clusters, which altogether may represent social aggregations. We suggest two complementary hypotheses to explain the blue whale female structure in the GC winter calving-feeding ground; one ecological, related to resource concentration, and a behavioural one, related to female social relationships.

From an ecological point of view, blue whale spatial distribution is linked to the distribution of euphausiids [75], [76], their dominant prey [77]. Therfore, the whale distribution observed in the GC may be the result of feeding area choices as found in other areas [75], [76]. In the GC, up to 30% of the blue whales recorded during the aerial surveys were observed feeding close to the surface and/or defecating (used as indirect clue of feeding behaviour), revealing that feeding is an important activity for blue whales, including lactating females, in this calving ground [26]. Mother-calf pair feeding behaviour in a Chilean summer ground has also been reported [78]. The southwestern GC is characterized by the presence of several coastal islands, where dense daytime surface swarms of the euphausiid *N. simplex* have been reported [66]. In particular, the north of the Carmen Island in front of Loreto and the area between the islands of San Jose and Espiritu Santo north of La Paz (24°50′- 24°36′) are characterized by the presence of a very high density of *N. simplex* during the winter [66], [79]. Each of these two areas corresponds to the spatial distribution of one of the two female genetic clusters. We hypothesized that, if there is a local segregation due to the heterogeneous environment, this could lead to some segregation among blue whale females and hence to the observed genetic divergence.

From a behavioural point of view, blue whale female-calf pairs prefer coastal waters, more protected from the strong dominant North wind and swell [26]. This female preference has been reported also in southern right whales [80], [81], [82] and humpback whales [83], [84]. In mammals, including baleen whales, there is evidence of strong female site fidelity [13], [85] and resulting in genetic divergence such as in grey whale [14]; elephants [74], ungulates [2] and sea lions [86]. In our study, the majority of mother-calf pairs (67%, n = 94) were sighted in San Jose-La Paz area (corresponding to the yellow cluster). Moreover, most (85% 17 out of 20) genotyped mother-calf pairs were observed in San Jose-La Paz area. Although mother-calf pairs are not restricted to this area, it appears to be more intensively used as a nursing area than Loreto area. In southern right whales (*Eubalaena australis*), mother-calf pair congregates in specific nursery grounds, and are segregated from males in winter grounds, possibly to reduce the occurrence of male harassment which can be fatal to the calves [80], [81], [87]. Contrasting with the right whales, foraging is an important activity for blue whales in the GC, thus mother-calf segregation could reduce not only male harassment, but also resource competition with males. If females that congregate in San Jose-La Paz area have a higher level of kinship than Loreto area, this could explain the divergence found between the two areas. Future kinship analysis among whale groups in each cluster may provide further insight into social structure and how it influences the population structure of blue whales in the GC. While it is known that some whales that use the GC in winter, are feeding off California in summer [24], fine-scale segregation among females has not been reported in this feeding ground. However, if site fidelity is a widespread behavior in female blue whales, segregation is likely to be observed in other areas, mainly among pregnant or late-nursing females in which energy loss should be minimized.

Female fine-scale genetic structure has potentially important consequences for conservation and management. Opportunities for kin selection may have a positive effect on calf survival. Blue whale females with calves, that need to implement efficient foraging strategies due to heavy energetic demands, may take advantage of the high seasonal productivity and calm waters of the southwestern GC islands, and share these areas with relatives. Mature females and calves survival has a dramatic impact on population demography, and, therefore, management policies tailored to the areas where mother-calf pairs are frequently sighted could be greatly beneficial for population viability. Moreover, female fine-scale genetic structure may influence the rate at which genetic diversity is lost through genetic drift. Our results suggest that in blue whales, the loss of genetic variation may be minimized by male mediated gene flow. In this context, a socially structured population, with at least two main groups with high levels of heterozygosity, may lead to a reduction of inbreeding, and of the effects of genetic drift.

## References

[1] Waples RS, Gaggiotti O (2006) What is a population? An empirical evaluation of some genetic methods for identifying the number of gene pools and their degree of connectivity. Mol Ecol 15: 1419–39. 16629801 10.1111/j.1365-294X.2006.02890.x

[2] Coltman DW, Pilkington JG, Pemberton JM (2003) Fine-scale genetic structure in a free-living ungulate population. Mol Ecol 12: 733–742. 12675828 10.1046/j.1365-294x.2003.01762.x

[3] Andersen E, Born EW, Dietz R, Haug T, Øien N, et al (2003) Genetic population structure of minke whales *Balaenoptera acutorostrata* from Greenland, the North East Atlantic and the North Sea probably reflects different ecological regions. Mar Ecol Prog Ser 247: 263–280.

[4] Bérubé M, Larsen F, Notarbartolo di Sciara G, Sears R, et al. Population genetic structure of North Atlantic, Mediterranean Sea and Sea of Cortez fin whales, *Balaenoptera physalus* (Linnaeus, 1758); analysis of mitochondrial and nuclear loci. Mol Ecol 7: 585–599. 10.1046/j.1365-294x.1998.00359.x 9633102

[5] Escorza-Treviño S, Pastene LA, Dizon EA (2004) Molecular analysis of the truei and dalli morphotypes of dall’s porpoise *Phocoenoides dalli* . J Mamm 85: 347–355.

[6] Mendez M, Rosembaum HC, Subramaniam A, Yackulic C, Bordino P (2010) Isolation by environmental distance in mobile marine species: molecular ecology of franciscana dolphins at their southern range. Mol Ecol 19: 2212–2228. 20465582 10.1111/j.1365-294X.2010.04647.x

[7] Patenaude NJ, Portway VA, Schaeff CM, Bannister JL, Best PB, et al (2007) Mitochondrial DNA Diversity and Population Structure among Southern Right Whales (*Eubalaena australis*). J Hered 98: 147–157. 17416933 10.1093/jhered/esm005

[8] Hoelzel AR (1998) Genetic structure of cetacean populations in simpatry, parapatry and mixed assemblages: implications for conservations and policy. J Hered 89: 451–458.

[9] Tiedemann R, Hardy O, Vekemans X, Milenkovitch M (2000) Higher impact of female than male migration on population structure in large mammals. Mol Ecol 9: 1159–1163. 10964235 10.1046/j.1365-294x.2000.00975.x

[10] Rosenbaum HC, Pomilla C, Mendez M, Leslie MS, Best PB, et al (2009) Population structure of humpback whales from their breeding grounds in the South Atlantic and Indian Oceans. PLoS ONE 4: e7318. 19812698 10.1371/journal.pone.0007318 PMC2754530

[11] Schaeff C, Kraus S, Brown MW, White B (1993) Assessment of the population structure of western North Atlantic right whales (*Eubalaena glacialis*) based on sighting and mtDNA data. Can J Zool 71: 339–345.

[12] Carroll EL, Childerhouse SJ, Christie M, Lavery S, Patenaude N, et al (2012) Paternity assignment and demographic closure in the New Zealand southern right whale. Mol Ecol 21: 3960–3973. 22726223 10.1111/j.1365-294X.2012.05676.x

[13] Valenzuela LO, Sironi M, Rowntree V, Seger J (2009) Isotopic and Genetic evidence for culturally inherited site fidelity to feeding ground in southern right whales (*Eubalaena australis*). Mol Ecol 18: 782–791. 19207250 10.1111/j.1365-294X.2008.04069.x

[14] Goerlitz DS, Urbán J, Rojas-Bracho L, Belson M, Schaeff CM (2003) Mitochondrial DNA variation among Eastern North Pacific grey whales (*Eschrichtius robustus*) on winter breeding grounds in Baja California. Can J Zool 81: 1965–1972.

[15] Frasier TR, Koroscil SM, White BN, Darling JD (2011) Assessment of population substructure in relation to summer feeding ground use in the eastern North Pacific gray whale. Endang Species Res 14: 39–48.

[16] Lyrholm T, Gyllensten U (1998) Global matrilineal population structure in sperm whales as indicated by mitochondrial DNA sequences. Proc R Soc 265: 1679–1684. 10.1098/rspb.1998.0488 PMC1689338 9753788

[17] Lyrholm T, Leimar O, Johanneson B, Gyllensten U (1999) Sex-biased dispersal in sperm whales: contrasting mitochondrial and nuclear genetic structure of global populations. Proc R Soc 266: 347–354. 10.1098/rspb.1999.0644 PMC1689695 10097396

[18] Sremba AL, Hancock-Hanser B, Branch TA, LeDuc RL, Baker CS (2012) Circumpolar Diversity and Geographic Differentiation of mtDNA in the Critically Endangered Antarctic Blue Whale (*Balaenoptera musculus intermedia*). PLoS ONE 7: e32579. 22412889 10.1371/journal.pone.0032579 PMC3296714

[19] Lockyer C, Brown SG (1981) The migration of whales. In: Aidley DJ editors. Animal migration Society for Experimental Biology. Cambridge: Univ Press. 105–137.

[20] Reilly SB, Bannister JL, Best PB, Brown M, Brownell Jr RL, et al. (2008) *Balaenoptera musculus* In: IUCN 2010. IUCN Red List of Threatened Species. Version 2010.4. <www.iucnredlist.org>. Downloaded on 27 september 2011.

[21] Calambokidis J, Barlow J (2004) Abundance of blue and humpback whale in the Eastern North pacific estimated by capture-recapture and line transect methods. Mar Mamm Sci 20: 63–85.

[22] Stafford KM, Nieukirk SL, Fox CG (1999) Low-frequency whale sounds recorded on hydrophones moored in the Eastern Tropical Pacific. J Acoustic Soc America 106: 3687–3698. 10.1121/1.428220 10615707

[23] Stafford KM, Nieukirk SL, Fox CG (2001) Geographical and seasonal variation of blue whale calls in the North Pacific. J Cetacean Res Manage 3: 65–76.

[24] Calambokidis J, Steiger GH, Cubbage JC, Balcom KC, Ewald C, et al (1990) Sightings and movements of blue whales off central California 1986–88 from photo-identification. Rep Int Whal Comm 12: 343–348.

[25] Bailey H, Mate BR, Palacios DM, Irvine L, Bograd SJ, et al.. (2009) Behavioural estimation of blue whale movements in the Northeast Pacific from state space model analysis of satellite tracks. Endang Species Res Preprint doi: 10.3354/esr00239.

[26] Gendron D (2002) Population Ecology of the Blue Whales, *Balaenoptera musculus,* of the Baja California Peninsula. Centro de Investigacion Cientifica y de Educacion Superior de Ensenada (CICESE), Mexico. Ph.D. Thesis.

[27] LeDuc RG, Dizon AE, Goto M, Pastene LA, Kato H, et al (2007) Patterns of genetic variation in Southern Hemisphere blue whales and use of assignment test to detect mixing on the feeding grounds. J Cetacean Res Manage 9: 73–80.

[28] Attard CRM, Beheregaray LB, Curt JC, Gill P, Jenner M, et al (2010) Genetic diversity and structure of blue whales (*Balaenoptera musculus*) in Australian feeding aggregations. Conserv Genet 11: 2437–2441.

[29] Gendron D, Ugalde De La Cruz A (2012) A new classification method to simplify blue whale photo-identification technique. J Cetacean Res Manage 12: 79–84.

[30] Wang IJ (2009) Fine-scale population structure in a desert amphibian: landscape genetics of the black toad (*Bufo exsul*). Mol Ecol 18: 3847–3856. 19708887 10.1111/j.1365-294X.2009.04338.x

[31] Nussey DH, Coltman DW, Coulson T, Kruuk LEB, Donald A, et al (2005) Rapidly declining fine-scale spatial genetic structure in female red deer. Mol Ecol 14: 3395–3405. 16156811 10.1111/j.1365-294X.2005.02692.x

[32] Roden GI (1964) Oceanographic aspects of the Gulf of California. In: van Andel TH, Shore J GG editors. Marine Geology of the Gulf of California.Tulsa: American Association of Petroleum Geologists Memoir. 30–58.

[33] Santamaría-del-Angel E, Alvarez-Borrego S, Müller-Karger F (1994) Gulf of California biogeographic regions based on coastal zone color scanner imagery. J Geoph Res 99: 7411–7421.

[34] Sears R, Williamson JM, Wenzel FW, Bérubé M, Gendron D, et al (1990) Photographic identification of the blue whale (*Balaenoptera musculus*) in the Gulf of St. Lawrence, Canada. Rep Int Whal Comm 12: 335–342.

[35] Lambertsen RH (1987) A biopsy system for large whales and its use for cytogenetics. J. Mamm 68(2): 443–445.

[36] Amos W, Hoelzel AR (1991) Long-term preservation of whale skin for DNA analysis. Rep Int Whal Comm 13: 99–103.

[37] Aljanabi SM, Martínez I (1997) Universal and rapid salt-extraction of high quality genomic DNA for PCR-base techniques. Nuc Acid Res 25: 4692–4693. 10.1093/nar/25.22.4692 PMC147078 9358185

[38] Bérubé M, Rew MB, Skaug H, Jorgensen H, Robbins J, et al (2005) Polymorphic microsatellite loci isolated from humpback whale, *Megaptera novaeangliae* and fin whale, *Balaenoptera physalus* . Conserv Genet 6: 631–636.

[39] Buchanan FC, Friesen MK, Littlejohn RP, Clayton JW (1996) Microsatellites from the beluga whale (*Delphinapterus leucas*). Mol Ecol 5: 571–575. 8794563

[40] Palsbøll PJ, Berube M, Jørgensen H (1999) Multiple Levels of Single-Strand Slippage at Cetacean Tri- and Tetranucleotide Repeat Microsatellite Loci. Genetics 151: 285–296. 9872967 10.1093/genetics/151.1.285 PMC1460447

[41] Bérubé M, Jørgensen H, McEwing R, Palsbøll PJ (2000) Polymorphic di-nucleotide microsatellite loci isolated from the humpback whale, *Megaptera novaeangliae* . Mol Ecol 9: 2181–2183. 11123643 10.1046/j.1365-294x.2000.105315.x

[42] Valsecchi E, Amos W (1996) Microsatellite markers for the study of cetacean populations. Mol Ecol 5: 151–156. 9147690 10.1111/j.1365-294x.1996.tb00301.x

[43] Amos W, Hoffman JI, Frodsham A, Zhang L, Best S, et al (2007) Automated binning of microsatellite alleles: problems and solutions. Mol Ecol Notes 7: 10–14.

[44] Van Oosterhout C, Hutchinson WF, Wills DP, Shipley P (2004) Micro-checker: software for identifying and correcting genotyping errors in microsatellite data. Mol Ecol Notes 4: 535–538.

[45] Bérubé M, Palsbøll PJ (1996) Identification of sex in cetaceans by multiplexing with three ZFX and ZFY specific primers. Mol Ecol 5: 283–287. 8673273 10.1111/j.1365-294x.1996.tb00315.x

[46] Excoffier L, Laval G, Schneider S (2005) Arlequin 3.0: an integrated software package for population genetics data analysis. Evol Bioinform Online 1: 47–50. PMC2658868 19325852

[47] Guo S, Thompson E (1992) Performing the exact test of Hardy-Weinberg proportion for multiple alleles. Biometrics 48: 361–372. 1637966

[48] Raymond M, Rousset F (1995) Genpop (version 1.2): Populations genetics software for exact test and ecumenicist. J Hered 86: 248–249.

[49] Rice WR (1989) Analyzing tables of statistical test. Evolution 43: 223–225. 28568501 10.1111/j.1558-5646.1989.tb04220.x

[50] Goudet J (1995) Fstat (version 1.2): a computer program to calculate F-statistics. J Hered 86: 485–486.

[51] Slatkin M (1995) A measure of population subdivision based on microsatellite allele frequencies. Genetics 139: 457–462. 7705646 10.1093/genetics/139.1.457 PMC1206343

[52] Wright S (1978) Evolution of the genetics populations. London: University of Chicago Press. 590 p.

[53] Benjamini Y, Yekutieli D (2001) The Control of the false discovery rate in multiple testing under dependency. Ann Statist 29: 1165–1188.

[54] Queller DC, Goodnight KF (1989) Estimating relatedness using genetic markers. Evolution 43: 258–275. 28568555 10.1111/j.1558-5646.1989.tb04226.x

[55] Lynch M, Ritland K (1999) Estimation of pairwise relatedness with molecular markers. Genetics 152: 1753–1766. 10430599 10.1093/genetics/152.4.1753 PMC1460714

[56] Bekoff M, Mech LD (1984) Computer Simulation: Simulation analyses of space use: Home range estimates, variability, and sample size. Behav Res Methods Instrum Comput 16: 32–37.

[57] Peakall R, Smouse PE (2006) Genalex 6: Genetic analysis in Excel. Population genetic software for teaching and research. Mol Ecol Notes 6: 288–295. 10.1093/bioinformatics/bts460 PMC3463245 22820204

[58] Pritchard JK, Matthew S, Donnely P (2000) Inference of population structure using multilocus genotype data. Genetics 155: 945–959. 10835412 10.1093/genetics/155.2.945 PMC1461096

[59] Guillot G, Leblois RL, Coulon A, Frantz AC (2009) Statistical methods in spatial genetics. Mol Ecol 18: 4734–4756. 19878454 10.1111/j.1365-294X.2009.04410.x

[60] Guillot G, Estoup A, Mortier F, Cosson JF (2005) A Spatial statistical model for landscape genetics. Gene Soc Amer 170: 1261–1280. 10.1534/genetics.104.033803 PMC1451194 15520263

[61] Worton BJ (1989) Kernel methods for estimating the utilization distribution in home-range studies. Ecology 70: 164–168.

[62] Rodgers AR, Carr AP, Smith L, Kie JG (2005) HRT: home range tools for ArcGIS. Ontario Ministry of Natural Resources Thunder Bay: Centre for Northern Forest Ecosystem Research.

[63] Latch EK, Dharmarajan G, Glaubitz JC, Rhodes Jr OE (2006) Relative performance of Bayesian clustering software for inferring population substructure and individual assignment at low levels of population differentiation. Conserv Genet 7: 295–302.

[64] Reilly SB, Thayer VG (1990) Blue whale (*Balaenoptera musculus*) distribution in the Eastern Tropical Pacific. Mar Mamm Sci 6: 265–277.

[65] Clapham PJ (2000) The humpback whale. In: Mann J, Connor RC, Tyack P, Whitehead H, editors. Cetacean society: field study of dolphins and whales. Chicago: University of Chicago Press. 173–196.

[66] Gendron D (1992) Population structure of daytime surface swam of *Nyctiphanes simplex* (Crustacea: Euphausiacea) in the Gulf of California, Mexico. Mar Ecol Prog Ser 87: 1–6.

[67] Brinton E, Townsend AW (1980) Euphausiids in the Gulf of California- the 1957 Cruises. Calcofi Rep. 21: 212–232.

[68] Francois O, Durand E (2010) Spatially explicit Bayesian clustering models in population genetics. Mol Ecol Res 10: 773–784. 10.1111/j.1755-0998.2010.02868.x 21565089

[69] Kalinowski ST (2011) The computer program STRUCTURE does not reliably identify the main genetic clusters within species: simulations and implications for human population structure. Heredity 106: 625–632. 20683484 10.1038/hdy.2010.95 PMC3183908

[70] Frantz AC, Cellina S, Krier A, Schley L, Burke T (2009) Using spatial Bayesian methods to determine the genetic structure of a continuously distributed population: clusters or isolation by distance? J App Ecol 46: 493–505.

[71] Safner T, Miller MP, McRae BH, Fortin MJ, Manel S (2011) Comparison of Bayesian clustering and edge detection methods for inferring boundaries in landscape genetics. Int J Mol Sci 12: 865–889. 21541031 10.3390/ijms12020865 PMC3083678

[72] Falush D, Stephens M, Pritchard JK (2003) Inference of population structure using multilocus genotype data: linked loci and correlated allele frequencies. Genetics 164: 1567–1587. 12930761 10.1093/genetics/164.4.1567 PMC1462648

[73] Rosenberg NA, Pritchard JK, Weber JL, Cann HM, Kidd KK, et al (2002) Genetic structure of human populations. Science 28: 2381–2385. 10.1126/science.1078311 12493913

[74] Okello JBA, Wittemyer G, Rasmussen HB, Arctander P, Nyakaana S, et al (2008) Effective population size dynamics reveal impacts of historic climatic events and recent anthropogenic pressure in African elephants. Mol Ecol 17: 3788–3799. 18643879 10.1111/j.1365-294X.2008.03871.x

[75] Branch TA, KM Stafford, Palacios DM, Allison C, Bannister JL, et al (2007) Past and present distribution, densities and movements of blue whales, *Balaenoptera musculus,* in the Southern Hemisphere and northern Indian Ocean. Mamm Rev 37: 116–175.

[76] Croll DA, Marinovic B, Benson S, Chavez FP, Black N, et al (2005) From wind to whales: trophic links in a coastal upwelling system. Mar Ecol Prog Ser 289: 117–130.

[77] Gaskin DE (1982) The ecology of whales and dolphins. London: Heinemann Educational Books. 459p.

[78] Hucke-Gaete R, Osman LP, Moreno C, Findlay KP, Ljungblad DK (2004) Discovery of a blue whale feeding and nursing ground in southern Chile. Proc R Soc B 271: 170–S173. 10.1098/rsbl.2003.0132 PMC1810017 15252974

[79] De Silva-Davila R, Palomares-Gacria JR (1998) Unusual larval growth production of *Nyctiphanes simplex* in Bahia de la Paz, Baja California Sur, Mexico. J Crust Biol 18: 490–498.

[80] Elwen SH, Best PB (2004a) Female southern right whale *Eubalaena australis:* Are there reproductive benefits associated with their coastal distribution off South Africa? Mar Ecol Prog Ser 269: 289–295.

[81] Elwen SH, Best PB (2004b) Environmental factors influencing the distribution of southern right whales (*Eubalaena australis*) on the southern coast of South Africa I: broad scale patterns. Mar Mamm Sci 20: 567–582.

[82] Thomas PO, Taber SM (1984) Mother-infant interaction and behavioral development in southern right whales, *Eubalaena australis* . Behaviour 88: 42–60.

[83] Clapham PJ (1996) The social and reproductive biology of humpback whales: and ecological perspective. Mamm Rev 37: 27–49.

[84] Craig AS, Herman LM (1997) Sex differences in site fidelity and migration of humpback whales (*Megaptera novaeangliae*) to the Hawaiian Islands. Can J Zool 75: 1923–1933.

[85] Baker CS, Palumbi SR, Lambertsenm RH, Weinrich MT, Calambokidisa J, et al (1990) The influence of seasonal migration on geographic distribution of mitochondrial DNA haplotypes in humpback whales. Nature 344: 238–240. 1969116 10.1038/344238a0

[86] Campbell RA, Gales NJ, Lento GM, Baker CS (2008) Islands in the sea: extreme female natal site fidelity in the Australian sea lion, *Neophoca cinerea* . Proc R Soc Biol Lett 4: 139–142. 10.1098/rsbl.2007.0487 PMC2412930 18042512

[87] Payne R (1986) Long term behavioral studies of the southern right whale (*Eubalaena australis*). Rep Int Whal Commn 10: 161–67.

