# Correction: Fine-Scale Population Structure of Blue Whale Wintering Aggregations in the Gulf of California

**DOI:** 10.1371/annotation/a9acdbf7-f6c0-4997-8232-7be417d300a3

**Published:** 2013-06-19

**Authors:** Paula Costa-Urrutia, Simona Sanvito, Nelva Victoria-Cota, Luis Enríquez-Paredes, Diane Gendron

The titles and legends for Figures 3 and 4 were incorrectly switched. The title and legend for Figure 3 should appear with Figure 4, and the title and legend for Figure 4 should appear with Figure 3. The figures themselves are in correct order. 

